# A Pain in the Neck of the Gallbladder: Mirizzi Syndrome

**DOI:** 10.7759/cureus.80423

**Published:** 2025-03-11

**Authors:** Rachael Hagen, Minh Thu T Nguyen, Stefan Thorarensen, Ashwin Pillai, Neil Parikh

**Affiliations:** 1 Internal Medicine, University of Connecticut Health, Farmington, USA; 2 Gastroenterology and Hepatology, University of Connecticut, Farmington, USA; 3 Medicine, Division of Gastroenterology and Hepatology, Hartford Hospital, Hartford, USA

**Keywords:** biliary stent, cholecystobiliary fistula, mirizzi syndrome, nonsurgical management, percutaneous cholecystostomy tube

## Abstract

Mirizzi syndrome (MS) occurs in cholelithiasis when gallstones obstruct the cystic duct or neck of the gallbladder, leading to compression of the common hepatic duct (CHD) and potentially causing ductal obstruction. This may result in the formation of a cholecystocholedochal fistula. Open cholecystectomy is the standard treatment. We present a case of MS in a heart transplant candidate, managed with stent placement and percutaneous cholecystostomy due to his high surgical risk. His course was complicated by stent migration. This case underscores how MS can be effectively managed with stent placement and percutaneous cholecystostomy.

## Introduction

Mirizzi syndrome (MS) is a rare cause of biliary obstruction, occurring when gallstones in the cystic duct or neck of the gallbladder compress the common hepatic duct (CHD) due to edema and inflammation. This compression can lead to bile duct erosion and the formation of a cholecystocholedochal or cholecystoduodenal fistula, resulting in obstructive jaundice [1]. Over a 23-year period, MS was identified in 0.18% of all cholecystectomies [2].

MS is often challenging to diagnose due to its nonspecific symptoms. Ultrasound is typically the first-line imaging modality, while magnetic retrograde cholangiopancreatography is considered the gold standard for diagnosis due to its high sensitivity and specificity. Once the level of obstruction is characterized, treatment is typically performed with endoscopic retrograde cholangiopancreatography (ERCP) [3].

The primary treatment for MS is cholecystectomy to remove the obstructing gallstone and prevent further bile duct damage. However, for patients who are not candidates for surgery, MS can be managed with percutaneous cholecystostomy for temporary drainage. Biliary stenting may also aid in preoperative biliary decompression, particularly in the presence of cholangitis. Stenting can also help surgeons identify the main duct involved when ducts are inflamed or scarred. We describe a case of an elderly male awaiting a heart transplant who developed acute cholecystitis due to MS, which was successfully treated with stent placement and percutaneous cholecystostomy as an alternative to cholecystectomy.

This article was previously presented as a meeting abstract at the 2024 American College of Gastroenterology Annual Meeting on October 27, 2024, in Philadelphia, PA.

## Case presentation

A 70-year-old male patient with nonischemic cardiomyopathy and heart failure with reduced ejection fraction (12%) presented with shortness of breath and was admitted for decompensated heart failure and cardiogenic shock. He was placed on an Impella® (Abiomed, Inc., Danvers, MA, United States) for temporary left ventricular mechanical support while awaiting a heart transplant. One month later, he developed right upper quadrant pain, accompanied by nausea and vomiting. He remained afebrile and hemodynamically stable. On physical examination, he exhibited jaundice, scleral icterus, and right upper quadrant tenderness to palpation. Laboratory results revealed leukocytosis and a marked rise in liver enzymes in a cholestatic pattern, with a normal lipase level, suggesting an obstructive process (Table 1).

**Table 1:** Complete blood work suggesting an obstructive cholestatic process. WBC: white blood cell, Hb: hemoglobin, Hct: hematocrit, BUN: blood urea nitrogen,  AST: aspartate aminotransferase, ALT: alanine transaminase, ALP: alkaline phosphatase.

Tests	Results three days prior	Results	Reference range
Complete blood count			
WBC	13.1	15.8	4,000-11,000/µL
Hb	12.9	13.1	13-18 g/dL
Hct	39	39.2	35%-46%
Platelet count	154	150	150-400 x 10^9^/L
Basic metabolic panel			
Sodium	132	136	135-145 mmol/L
Potassium	4.4	4.1	3.5-5 mmol/L
Chloride	101	104	98-107 mmol/L
BUN	18	11	10-50 mg/dL
Creatinine	1	0.9	0.5-1.3 mg/dL
Calcium	8.6	9	8.5-10.5 mg/dL
Liver function tests			
Total bilirubin	0.5	5.1	0.3-1.0 mg/dL
Direct bilirubin	<0.2	4.8	<0.3 mg/dL
AST	46	548	13-39 U/L
ALT	59	526	7-52 U/L
ALP	98	684	34-104 U/L
Lipase	NA	35	13-60 U/L

**Figure 1:**
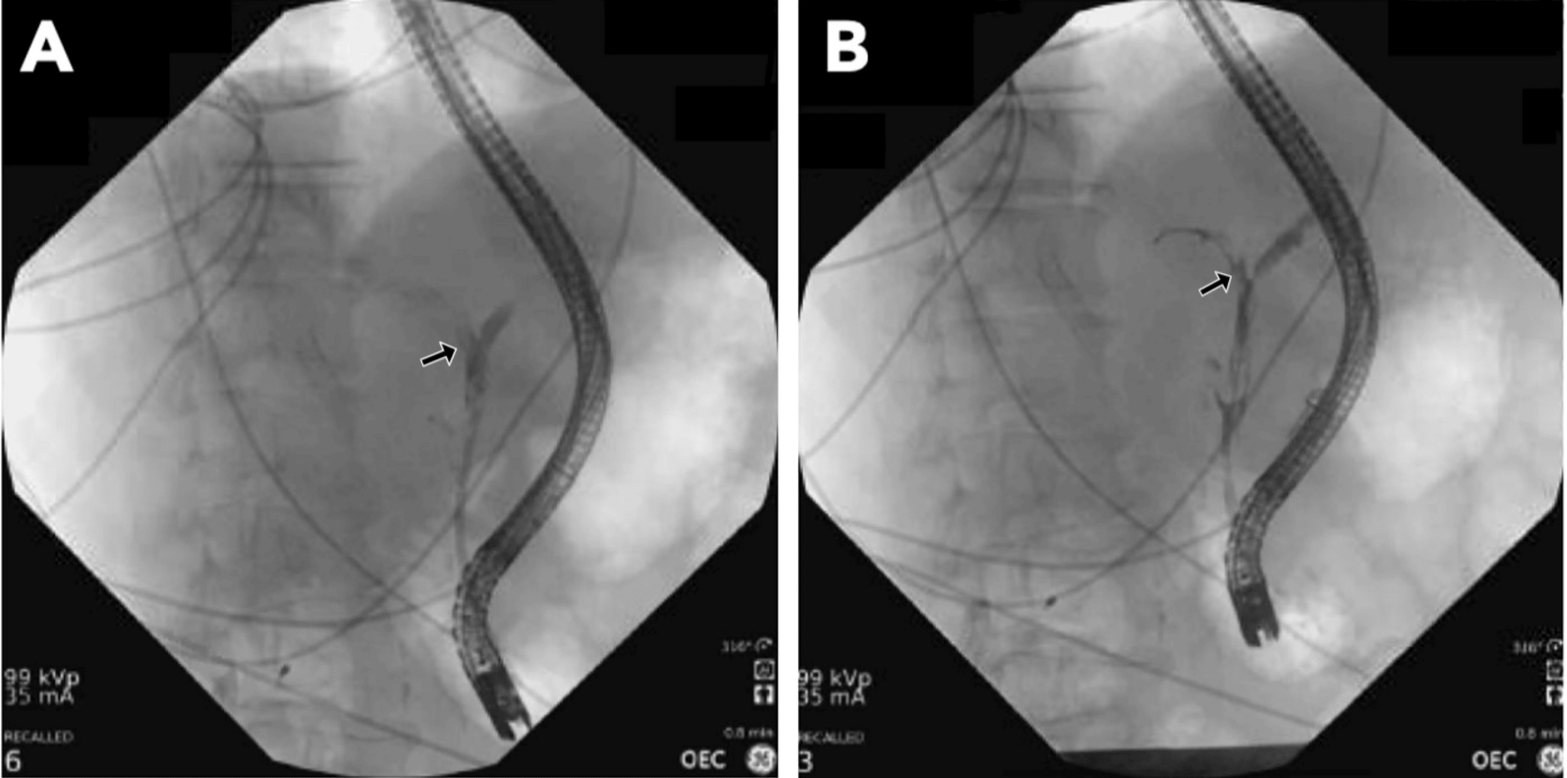
(A, B) Endoscopic retrograde cholangiopancreatography revealed localized stenosis in the middle third of the common bile duct, measuring 15 mm, consistent with Mirizzi syndrome type II (arrow), according to the Csendes classification system. Two plastic biliary stents were then placed.

Right upper quadrant ultrasound revealed cholelithiasis without signs of cholecystitis or biliary duct dilation. Given a high clinical suspicion for a hepatobiliary process, abdominal computed tomography (CT) was performed, which showed biliary ductal dilation, gallbladder wall thickening, pericholecystic fluid, and cholelithiasis. These findings were consistent with acute cholecystitis. ERCP identified a cholecystocholedochal fistula in the middle third of the common bile duct (CBD), along with a stone in the cystic duct, consistent with Mirizzi syndrome type II (Table 2). Plastic stent placement was required (Figure 1A, 1B).

**Table 2:** Csendes classification of the Mirizzi syndrome. This table is published under a Creative Commons License: Csendes et al. (1989) [4], Klekowskiet al. (2021) [5].

Classification	Type I	Type II	Type III	Type IV
Description	External compression of the bile duct	Cholecystobiliary fistula involving less than one-third of the bile duct wall erosion	Cholecystobiliary fistula involving one-third to two-thirds of the bile duct wall erosion	Cholecystobiliary fistula involving more than two-thirds of the bile duct wall erosion

A plastic stent was chosen over a metallic stent for easier removal/replacement and lower infection risk. Due to his poor candidacy for cholecystectomy, the patient underwent percutaneous cholecystostomy tube placement and was advised to return for follow-up ERCP in three months for stent removal.

Two weeks later, the patient developed nausea, vomiting, and right upper quadrant pain, accompanied by rising liver enzymes. An abdominal CT scan revealed a dislodged biliary duct stent in the small bowel in the left upper quadrant, along with biliary ductal dilation (1.3 cm) due to gallbladder sludge and stones. He underwent repeat ERCP, which identified significant clot burden with hemobilia, requiring sweeping of the biliary tree and placement of a covered metal stent in the CBD.

His symptoms improved, the migrated plastic stent passed uneventfully, and his liver enzymes normalized. He did not develop cholangitis or other complications. A repeat ERCP in three months was planned for stent removal. However, before stent removal could take place, he died from various postoperative cardiac complications after receiving a heart transplant two months after presentation.

## Discussion

MS occurs when gallstones obstruct the CHD, potentially leading to bile duct erosion and the formation of an obstructed duct and/or cholecystocholedochal fistula. Risk factors include a tortuous cystic duct, low insertion of the cystic duct into the CBD, and a thin gallbladder wall [4]. Several classification systems for MS exist, with the Csendes classification system being the most widely used (Table 2) [4,5]. This patient was diagnosed with MS type II, characterized by a cholecystobiliary fistula involving less than one-third of the circumference of the CHD.

The pathophysiology of MS involves gallstones causing gallbladder distention and thickening, which leads to inflammation of the cystic duct and sometimes the gallbladder, as well as external compression of the CHD. If left untreated, chronic inflammation may result in bile duct wall necrosis and the subsequent formation of a cholecystobiliary fistula [6].

Optimal outcomes for MS depend on timely evaluation and intervention, typically involving ERCP for stone retrieval and stent placement. Early management is essential to prevent complications, such as bile duct injury. Failure to diagnose MS preoperatively is associated with increased morbidity, mortality, complications, and reoperation rates [6]. Open cholecystectomy remains the gold standard treatment, although laparoscopic approaches are increasingly utilized, despite the absence of internationally accepted guidelines [7]. Notably, the conversion rate from laparoscopy to open laparotomy is high (34.09%) [1], underscoring the importance of careful surgical risk assessment, particularly in patients who may not tolerate open procedures.

Our patient was unable to undergo surgery due to high-risk comorbidities. The use of percutaneous cholecystostomy and stenting to manage MS is rare and typically reserved for elderly patients with severe comorbidities that significantly impact their prognosis [8]. Stents are generally employed as a temporizing measure before surgery but have been reported as a successful definitive treatment in patients with MS type II who are poor surgical candidates [9]. Although the case was complicated by stent migration, the approach successfully achieved interim management. We acknowledge the limitations of this case, given the patient’s passing two months later. However, it underscores a real-world scenario where less invasive options may be necessary for patients who are not surgical candidates.

Recently, alternative endotherapies to percutaneous drainage of the gallbladder have emerged for treating MS in patients who are poor surgical candidates. These include endoscopic ultrasound (EUS)-guided cholecystogastrostomy, cholangioscopy-directed electrohydraulic lithotripsy, laser lithotripsy (LL), and extracorporeal shock wave lithotripsy (ESWL). EUS-guided cholecystogastrostomy is preferred over percutaneous cholecystostomy as it provides internal gallbladder drainage and obstruction relief without the need for a persistent indwelling catheter. Cholangioscopy-guided LL has demonstrated a high rate of single-session ductal clearance rate of 94% in a case series of MS patients [10]. Similarly, ESWL has shown favorable outcomes by effectively fragmenting stones, which are then extracted endoscopically [11]. Although these techniques were not available at the time, they might have served as effective alternative treatment options, especially if percutaneous cholecystostomy had failed. This case highlights the potential for successful nonsurgical management of MS in patients with significant comorbidities.

## Conclusions

MS is a rare but significant complication of cholelithiasis that requires timely recognition and intervention. While open cholecystectomy remains the standard treatment, alternative management strategies, such as ERCP with stenting, percutaneous drainage, and emerging endoscopic techniques, provide viable options for high-risk surgical candidates. This case emphasizes the importance of individualized treatment approaches, especially in patients with significant comorbidities. Advances in cholangioscopy-directed lithotripsy and ESWL may further expand nonsurgical treatment modalities. Continued research and refinement of minimally invasive techniques will be crucial in optimizing outcomes for patients with MS who are unsuitable for traditional surgical intervention.
